# Bionomic characterization of *Anopheles* mosquitoes in the Ethiopian highlands and lowlands

**DOI:** 10.1186/s13071-024-06378-3

**Published:** 2024-07-16

**Authors:** Endashaw Esayas, Muluken Assefa, Adam Bennett, Edward Thomsen, Steven Gowelo, Elodie Vajda, Asefaw Getachew, Temesgen Ashine, Abebaw Yeshaneh, Fikregabrail Aberra Kassa, Mulugeta Demisse, Henry Ntuku, Hunduma Dinka, Lemu Golassa, Neil F. Lobo, Endalamaw Gadisa

**Affiliations:** 1 Malaria and Neglected Tropical Diseases Research Division, Armauer Hansen Research Institute, Addis Ababa, Ethiopia; 2 Aklilu Lemma Institute of Pathobiology, Addis Ababa University, Addis Ababa, Ethiopia; 3 PATH Malaria Control and Elimination Partnership in Africa (MACEPA), Seattle, USA; 4 Malaria Elimination Initiative, University of California San Francisco, San Francisco, USA; 5 PATH Malaria Control and Elimination Partnership in Africa (MACEPA), Addis Ababa, Ethiopia; 6 West Gondar Zone Health Department, Amhara Regional Health Bureau, Metema, Ethiopia; 7 Department of Applied Biology, School of Applied Natural Science, Adama Science and Technology University, Adama, Ethiopia; 8 Eck Institute for Global Health, University of Notre Dame, Notre Dame, Indiana USA

**Keywords:** *Anopheles* species, Malaria, Vector behaviors, Highland, Lowland, Seasonal migrant workers, Resident population, Ethiopia

## Abstract

**Background:**

The protective effectiveness of vector control in malaria relies on how the implemented tools overlap with mosquito species-specific compositions and bionomic traits. In Ethiopia, targeted entomological data enabling strategic decision-making are lacking around high-risk migrant worker camps in the lowlands and resident communities in the highlands—resulting in suboptimal malaria control strategies for both populations. This study investigates spatial and temporal mosquito behavior, generating baseline evidence that will improve malaria control for both migrant workers in the lowlands and their home communities in the highlands.

**Methods:**

Hourly Centers for Disease Control and Prevention (CDC) light trap collections were performed indoors and outdoors during the peak (October to December 2022) and minor (March to May 2023) malaria transmission seasons. These seasons coincide with the post-long rain and post-short rain seasons, respectively. Eight resident households were sampled from each of four villages in the highlands and eight households/farm structures on and near farms in four villages in the lowlands. The sampling occurred between 18:00 and 06:00. Spatiotemporal vector behaviors and hourly indoor and outdoor mosquito capture rates, used as a proxy for human biting rates, were calculated for overall catches and for individual species. Adult mosquitoes were identified using morphological keys, and a subset of samples were confirmed to species by sequencing ribosomal DNA internal transcribed spacer region 2 (ITS2) and/or mitochondrial DNA cytochrome *c* oxidase subunit 1 (Cox1).

**Results:**

In the highlands, 4697 *Anopheles* mosquitoes belonging to 13 morphologically identified species were collected. The predominant species of *Anopheles* identified in the highlands was *An. gambiae* sensu lato (s.l.) (*n* = 1970, 41.9%), followed by *An. demeilloni* (*n* = 1133, 24.1%) and *An. cinereus* (*n* = 520, 11.0%). In the lowland villages, 3220 mosquitoes belonging to 18 morphological species were collected. *Anopheles gambiae* s.l. (*n* = 1190, 36.9%), *An. pretoriensis* (*n* = 899, 27.9%), and *An. demeilloni* (*n* = 564, 17.5%) were the predominant species. A total of 20 species were identified molecularly, of which three could not be identified to species through comparison with published sequences. In highland villages, the indoor *Anopheles* mosquito capture rate was much greater than the outdoor rate. This trend reversed in the lowlands, where the rate of outdoor captures was greater than the indoor rate. In both highlands and lowlands, *Anopheles* mosquitoes showed early biting activities in the evening, which peaked between 18:00 and 21:00, for both indoor and outdoor locations.

**Conclusions:**

The high diversity of *Anopheles* vectors and their variable behaviors result in a dynamic and resilient transmission system impacting both exposure to infectious bites and intervention effectiveness. This creates gaps in protection allowing malaria transmission to persist. To achieve optimal control, one-size-fits-all strategies must be abandoned, and interventions should be tailored to the diverse spatiotemporal behaviors of different mosquito populations.

**Graphical Abstract:**

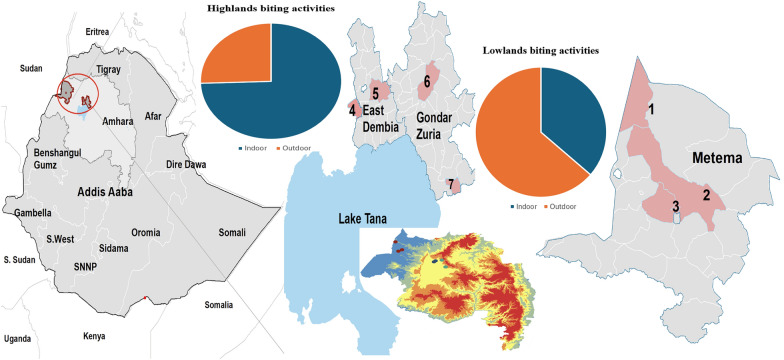

## Background

In Ethiopia, malaria remains a public health challenge that causes significant morbidity and mortality [1]. The disease is endemic in approximately 68% of the country, with 60% of the population at risk. Malaria transmission in Ethiopia is generally unstable and heterogeneous due to diverse eco-topographies and local weather patterns [2–4]. The highest risk for malaria infection is in the lowlands and in the west of the country along the border between Sudan and South Sudan, with geographies fringing the highlands being prone to frequent outbreaks [5]. Malaria is present up to 2000 m above sea level (masl); however, several pockets up to 2400 masl have micro-epidemiological conditions that support malaria transmission [6, 7].

The highlands surrounding Lake Tana [8], along with agricultural development corridors in adjacent lowland areas, are recognized as high-risk areas for malaria transmission [9]. Seasonal migrant workers who move from the lower-risk highlands to malaria-endemic lowlands for labor in farms and for other job opportunities [10] usually reside in open and temporary sleeping structures, thereby increasing exposure to infectious bites [9]. This both results in the exposure of less immune highland populations to malaria but also represents a population that continuously moves parasites back to the highlands [10]. Consequently, highland communities are vulnerable to frequent outbreaks due to the presence of primary and secondary malaria vectors along with introduced malaria sustaining the parasite reservoir [11].

Malaria control efforts are threatened by competent and abundant, anthropophilic and anthropophagic vectors [12] that demonstrate resistance to World Health Organization (WHO)-recommended insecticides [13, 14]. In Ethiopia, *Anopheles arabiensis* is the primary malaria vector [15] while *An. pharoensis*, *An. funestus* sensu lato (s.l.), and *An. nili* s.l. are considered secondary human malaria transmission vectors [3, 15–17]. The vector composition and distribution depend on the topography and climate of the country. In the highland areas of the country, *An. arabiensis*, *An. christyi*, *An. demeilloni*, *An. coustani*, and *An. cinereus* have been documented [6–8], whereas *An. arabiensis*, *An. funestus* s.l., *An. demeilloni*, and *An. pharoensis* are found in the lowlands [7, 18, 19]. Recently, *An. stephensi* was detected in the eastern part of Ethiopia, with documented expansion to other parts of the country [20]. This invasive vector has been reported to be highly permissive to *Plasmodium vivax* and *Plasmodium falciparum* in Ethiopia [21].

The presence of a diverse set of malaria vectors is indicative of a dynamic and resilient transmission system due to multiple species-specific bionomic traits that can respond to intervention strategies. Understanding this vector species diversity and their relevant behaviors enables strategic decision-making that necessitates the optimal selection and implementation of interventions that map to these behavioral traits [22]. To better understand the entomological drivers of transmission, this study sought to characterize the composition and bionomic traits of *Anopheles* mosquitoes in both high-transmission lowlands and vulnerable highlands, and in high-risk migrant and resident populations.

## Methods

### Study sites

**Fig. 1:**
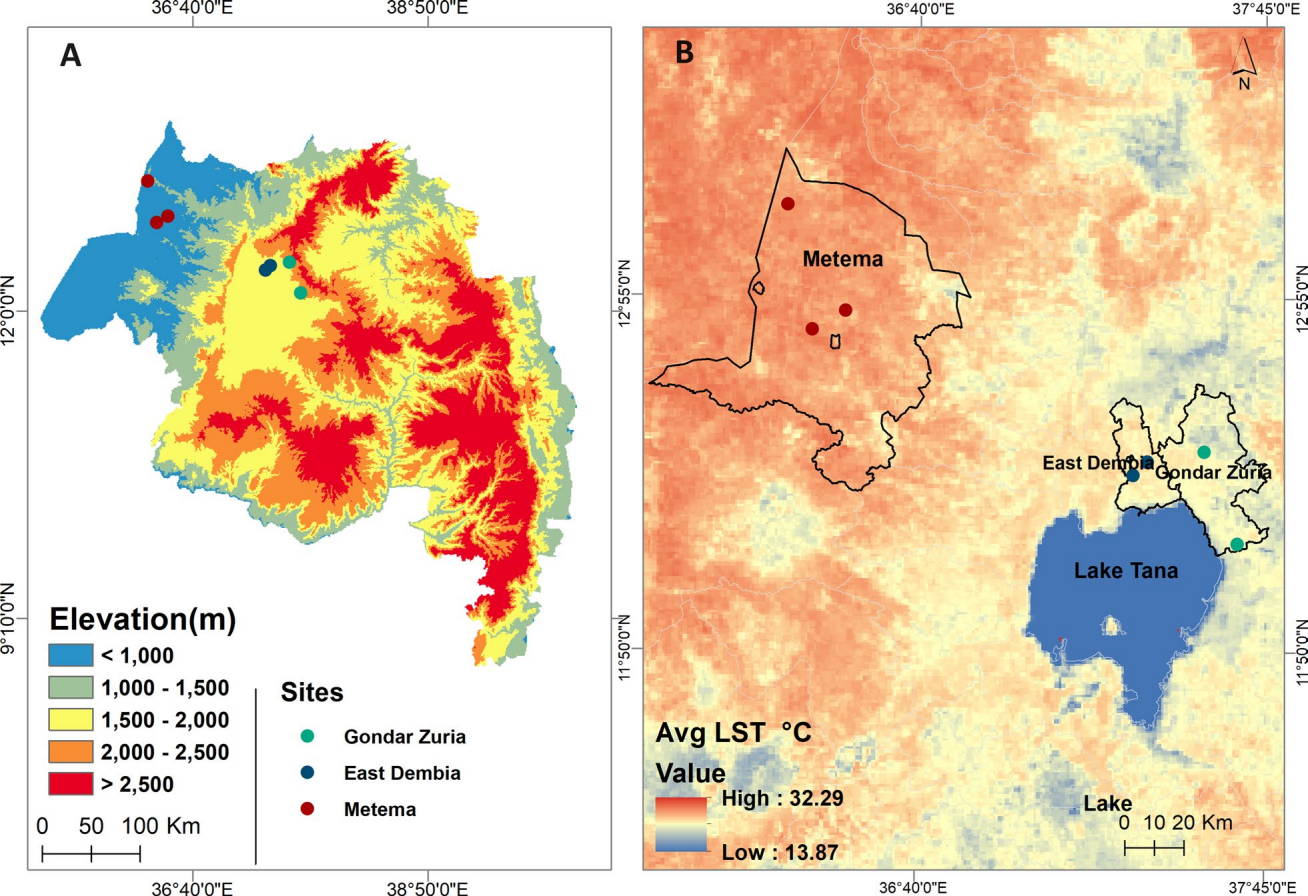
Map depicting the study sites of Gondar Zuria and East Dembia from the highlands and Metema from the lowlands. **A** Elevation map of the Amhara regional state with study sites. **B** Average land surface temperature (LST) map

Entomological surveys were conducted in Gondar Zuria and East Dembia districts in the highlands, and Metema district in the lowlands (Fig. 1A). The selection of the districts and sites was based on the known presence of seasonal migrant workers and historical high malaria incidence. Gondar Zuria and East Dembia are the permanent highland residential areas for the migrant workers, while Metema is their temporary seasonal destination in the lowlands. Gondar Zuria is located on the northeast edge of Lake Tana. The altitude ranges from 1800 to 2770 masl. East Dembia is in the central Gondar administrative zone, bordering Lake Tana to the south, and has similar geography and malaria epidemiology to Gondar Zuria [8]. The altitude of East Dembia ranges between 1500 and 2600 masl. The total population is estimated at 248,807 for Gondar Zuria and 307,967 for East Dembia [23]. Metema, one of the development corridors in the northwestern Ethiopian lowlands, has an average altitude of 750 (500–1000) masl. The district is one of nine agricultural investment districts, with a total permanent resident population of 154,618. A large number of migrant workers move to the district each year during the planting, weeding, and harvesting seasons [1]. East Dembia and Gondar Zuria experience two rainy seasons: long rains (June–September) and short rains (February–March). East Dembia is slightly warmer (14 °C min to 26 °C max), while Gondar Zuria is cooler (12.7 °C min to 25.1 °C max). Metema has a distinct dry winter tropical climate with year-round warmth (18 °C min to 29.4 °C max) (Fig. 1B).

**Fig. 2:**
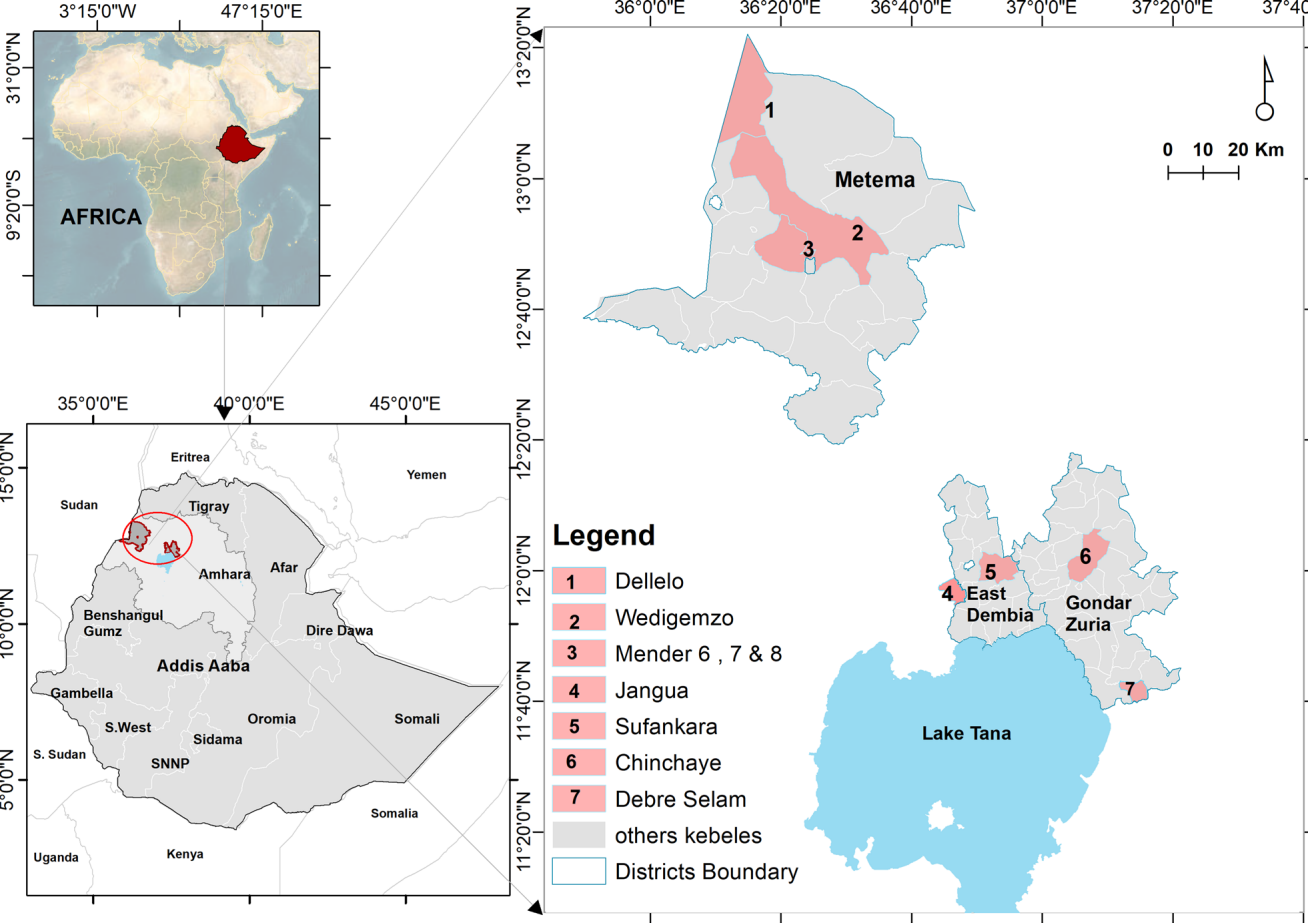
Map of Ethiopia showing the locations and administrative districts included in this study

The villages of Chinchaye and Debre Selam were selected from the Gondar Zuria district, while the villages of Jangua and Sufankara were selected from the East Dembia district. From the lowlands, two seasonal migrant worker camps (Dellelo-one and Dellelo-two farm areas) and two villages from the resident population sites (Wedigemzo and Mender-sidist) located within 30 km of the farm areas were selected (Fig. 2). The two highland districts harbor several types of mosquito larval habitats, including artificial pits, drainage canals, swamps, river pools, and seasonal rivers such as the Megech River. The Megech River drains into Lake Tana and persists until the end of December, where it serves as a water source for the communities that border it. The Guwang and other seasonal rivers also run through Metema district, resulting in numerous riverine pools that support vector populations.

### Entomological sampling methods

Hourly indoor and outdoor collections using Centers for Disease Control and Prevention light traps (CDC LT) were conducted during the peak (October to December 2022) and minor (March to May 2023) malaria transmission seasons. These seasons coincide with the post-long rain period (June–September), i.e. peak season, and post-short rain period (February–March), i.e. minor season in the study areas. In the highlands, a total of 32 households were selected across four villages, with eight households chosen as sentinel sites in each village. Each structure was sampled for 13 days during the peak transmission season and 10 days during the minor season. This resulted in a total of 208 collection nights during the peak season (52 nights per village) and 160 collection nights during the minor season (40 nights per village).

In the lowlands, both resident sentinel households and seasonal migrant worker structures were sampled. Two resident villages were sampled with eight sentinel households per village (total of 16 households). Two farm sites were sampled with eight sentinel migrant worker structures per farm (total of 16 structures). Similar to the highlands, each structure was sampled for 13 days during the peak season and 10 days during the minor season. In total, there were 104 collection nights during the peak season and 80 collection nights during the minor season across both resident and migrant farm worker populations. The sum of resident sentinel households and seasonal migrant worker structures resulted in a total of 208 and 160 collection nights across the lowlands in the peak and minor seasons, respectively. Overall, a total of 368 collection nights were conducted in both the highlands and lowlands, with 208 nights occurring during the peak season and 160 nights during the minor season.

Hourly CDC LT collections extended from 18:00 to 06:00. In each selected structure, the traps were positioned indoors (near the sleeping area of the inhabitants) and outdoors (~ 10 m away from the house entrance). The CDC LT collection cup was changed hourly by a two-person entomology team per house. The entomology teams were closely supervised to verify the timing and consistency of mosquito collections. Captured mosquitoes were stored in individual labeled collection cups and killed by freezing or alcohol. After sorting to sex and genus, female *Anopheles* mosquitoes were individually preserved in Eppendorf tubes with silica gel, labeled with date, household identification (ID), location, and hour of collection, and stored for further analysis.

### Molecular processing of *Anopheles* mosquitoes

Morphological identification of *Anopheles* mosquitoes was performed using the key developed by Gillies and Coetzee [24]. A subset of randomly chosen specimens (*n* = 663) from all morphologically identified species were sequenced at the ribosomal DNA internal transcribed spacer region 2 (ITS2) and/or the mitochondrial DNA cytochrome *c* oxidase subunit 1 (Cox1) locus, as previously described by Laurent et al. [25]. All selected samples were first amplified with ITS2-specific primers (ITS2A and ITS2B) [26]. Samples that failed to amplify (conclusive for species identification) or those with novel ITS2 sequences were subsequently amplified with Cox1 primers for further clarification [25]. Amplification of mitochondrial DNA Cox1 was conducted by adapting the procedure described by Folmer et al. [27] using light cycle oil (LCO) and heavy cycle oil (HCO) primers.

### Sequence analysis and species identification

Raw ITS2 sequences were initially assembled and checked for quality, then divided into "species groups" based on single-nucleotide polymorphisms (SNPs) with a minimum identity threshold of 98%. Cox1 sequences underwent a similar process with a final minimum match of 95% due to expected higher mitochondrial divergence. Both ITS2 and Cox1 sequences were compared to databases (NCBI nr and BOLD [28] for Cox1) for species identification. Analyses considered neither morphology nor single sequence contigs. High sequence identity (99% or greater) to voucher specimens was the primary confirmation method. When either ITS2 or Cox1 alone lacked significant voucher matches, results from both were combined. Manual inspection ensured proper sequence assembly and mitigated the impact of insertions/deletions on identity scores. Finally, the manually examined consensus sequences of each group were compared (BLASTn) to the NCBI nr database for definitive species identification whenever possible. This combined approach using sequence similarity and voucher specimen presence allowed for robust species identification.

### Data management and statistical analysis

Data were collected electronically using tablets preloaded with forms designed in REDCap software version 11.0.3 [29]. After collection, the data were uploaded to a secure server. Following download, the data were cleaned and formatted in Microsoft Excel (Microsoft Corp., Redmond, WA, USA). Statistical analysis employed a combination of Microsoft Excel and Stata software (version 17; StataCorp LLC, College Station, TX, USA). Only *Anopheles* mosquito identifications confirmed to the species level were included. Spatiotemporal vector behaviors and hourly indoor and outdoor capture rates (as a proxy for human biting rates) were determined for all *Anopheles* species and individual species during the collection period from 18:00 to 06:00. This aimed to identify overall and species-specific biting trends, including biting times, peak biting times, and preferred biting locations (indoor or outdoor) throughout the night. CDC LT captures were reported as mosquitoes per trap per night (mtn) or mosquitoes per trap per hour (mth) for location (indoors and outdoors) and site. Indoor and outdoor mtn means were compared using non-parametric Wilcoxon signed-rank tests. To estimate the relative abundance of each *Anopheles* mosquito species at a specific site, the number of captures for that species was divided by the total number of mosquitoes captured at that site. Additionally, the proportion of each *Anopheles* species relative to the total collection at each site was calculated.

## Results

### Vector species composition and relative abundance

#### The highland villages

In the highlands, a total of 4697 *Anopheles* mosquitoes were captured over 368 collection nights, representing 13 morphologically identified *Anopheles* species. *Anopheles gambiae* s.l. was the most abundant species found (*n* = 1970; 41.9%), followed by *An. demeilloni* (*n* = 1133; 24.1%) and *An. cinereus* (*n* = 520; 11.1%) (Table 1).

**Table 1:** *Anopheles* mosquito species composition and relative abundance in the four highland villages of the Gondar Zuria and East Dembia districts, northwestern Ethiopia

*Anopheles* species (Morphological)	Highland villages						
	Gondar Zuria district		East Dembia district		Highland total		
	Chinchaye village *n* (%)	Debre Selam village *n* (%)	Jangua village *n* (%)	Sufankara village *n* (%)	Total number of *Anopheles* catches	Relative abundance of species	Mean number of *Anopheles* per trap per night
*An. gambiae* s.l.	657 (43.28)	440 (41.90)	721 (41.94)	152 (37.07)	1970	41.94	5.35
*An. demeilloni*	313 (20.62)	352 (33.52)	329 (19.14)	139 (33.90)	1133	24.12	3.08
*An. cinereus*	428 (28.19)	24 (2.29)	21 (1.22)	47 (11.46)	520	11.07	1.41
*An. garnhami*	29 (1.91)	77 (7.33)	122 (7.10)	20 (4.88)	248	5.28	0.67
*An. pharoensis*	8 (0.53)	35 (3.33)	182 (10.59)	2 (0.49)	227	4.83	0.62
*An. christyi*	26 (1.71)	4 (0.38)	157 (9.13)	3 (0.73)	190	4.05	0.52
*An. salbaii*	5 (0.33)	41 (3.90)	68 (3.96)	34 (8.29)	148	3.15	0.40
*An. dancalicus*	20 (1.32)	16 (1.52)	34 (1.98)	5 (1.22)	75	1.60	0.20
*An. pretoriensis*	23 (1.52)	16 (1.52)	11 (0.64)	5 (1.22)	55	1.17	0.15
*An. coustani*	0 (0.00)	6 (0.57)	47 (2.73)	0 (0.00)	53	1.13	0.14
*An. fuscivenosus*	8 (0.53)	38 (3.62)	0 (0.00)	0 (0.00)	46	0.98	0.13
*An. tenebrosus*	0 (0.00)	1 (0.10)	27 (1.57)	2 (0.49)	30	0.64	0.08
*An. natalensis*	1 (0.07)	0 (0.00)	0 (0.00)	1 (0.24)	2	0.04	0.01
Total	1518 (32.32)	1050 (22.35)	1719 (36.60)	410 (8.73)	4697		

*n* number of *Anopheles* catches per village, *%* proportion of *Anopheles* catches per village

#### The lowland villages

In the lowlands, a total of 3220 *Anopheles* mosquitoes were captured over 368 trapping nights. Of these, 18 *Anopheles* species were identified morphologically, with one unknown specimen. *Anopheles gambiae* s.l. (*n* = 1190; 36.9%), *An. pretoriensis* (*n* = 899; 27.9%), and *An. demeilloni* (*n* = 564; 17.5%) were the most abundant species across all lowland villages. About 78.4% of the total *Anopheles* collected in the lowlands were from Wedigemzo village (Table 2).

**Table 2:** *Anopheles* mosquito species composition and relative abundance in the four villages of the lowland, seasonal migrant workers camps, and resident population villages, Metema, northwestern Ethiopia

*Anopheles* species (morphological)	Lowlands—seasonal migrant workers camps			Lowland villages			Lowland total		
				Lowlands—resident population villages					
	Dellelo-one village *n* (%)	Dellelo-two village *n* (%)	SMW *N* (%)	Mender-sidist village *n* (%)	Wedigemzo village *n* (%)	RP *N* (%)	Total number of *Anopheles* catches	Relative abundance of species	Mean number of *Anopheles* per trap per night
*An. gambiae* s.l.	177 (73.44)	231 (79.66)	408 (76.84)	106 (64.24)	676 (26.78)	782 (29.08)	1190	36.96	3.23
*An. pretoriensis*	27 (11.20)	28 (9.66)	55 (10.36)	22 (13.33)	822 (32.57)	844 (31.39)	899	27.92	2.44
*An. demeilloni*	15 (6.22)	6 (2.07)	21 (3.96)	21 (12.73)	522 (20.68)	543 (20.19)	564	17.52	1.53
*An. fuscivenosus*	6 (2.49)	12 (4.14)	18 (3.39)	5 (3.03)	197 (7.81)	202 (7.515)	220	6.83	0.60
*An. dancalicus*	1 (0.41)	4 (1.38)	5 (0.94)	3 (1.82)	64 (2.54)	67 (2.49)	72	2.24	0.20
*An. natalensis*	2 (0.83)	1 (0.34)	3 (0.56)	0 (0.00)	68 (2.69)	68 (2.53)	71	2.20	0.19
*An. rufipes*	3 (1.24)	2 (0.69)	5 (0.94)	0 (0.00)	66 (2.61)	66 (2.45)	71	2.20	0.19
*An. coustani*	0 (0.00)	0 (0.00)	0 (0.00)	0 (0.00)	52 (2.06)	52 (1.93)	52	1.62	0.14
*An. salbaii*	3 (1.24)	1 (0.34)	4 (0.75)	1 (0.61)	20 (0.79)	21 (0.78)	25	0.78	0.07
*An. schwetzi*	0 (0.00)	0 (0.00)	0 (0.00)	1 (0.61)	16 (0.63)	17 (0.63)	17	0.53	0.05
*An. pharoensis*	3 (1.24)	2 (0.69)	5 (0.94)	1 (0.61)	6 (0.24)	7 (0.26)	12	0.37	0.03
*An. garnhami*	0 (0.00)	0 (0.00)	0 (0.00)	2 (1.21)	3 (0.12)	5 (0.19)	5	0.16	0.01
*An. hervyi*	0 (0.00)	1 (0.34)	1 (0.19)	1 (0.61)	3 (0.12)	4 (0.15)	5	0.16	0.01
*An. tenebrosus*	1 (0.41)	0 (0.00)	1 (0.19)	2 (1.21)	2 (0.08)	4 (0.15)	5	0.16	0.01
*An. rhodesiensis*	2 (0.83)	0 (0.00)	2 (0.38)	0 (0.00)	2 (0.08)	2 (0.07)	4	0.12	0.01
*An. maculipalpis*	0 (0.00)	0 (0.00)	0 (0.00)	0 (0.00)	3 (0.12)	3 (0.11)	3	0.09	0.01
*An. longipalpis*	1 (0.41)	2 (0.69)	3 (0.56)	0 (0.00)	0 (0.00)	0 (0.00)	3	0.09	0.01
*An. christyi*	0 (0.00)	0 (0.00)	0 (0.00)	0 (0.00)	1 (0.04)	1 (0.04)	1	0.03	0.00
Unknown	0 (0.00)	0 (0.00)	0 (0.00)	0 (0.00)	1 (0.04)	1 (0.04)	1	0.03	0.00
Total	241 (7.48)	290 (9.01)	531	165 (5.12)	2524 (78.38)	2689	3220		

*n* number of *Anopheles* catches per village, *%* proportion of *Anopheles* catches per village or population, *N* total number of *Anopheles* catches per population (SMW/RP), *RP* resident population, *SMW* seasonal migrant workers

### Molecular species determination of *Anopheles*

Sequencing of ITS2 and/or Cox1 regions in 663 *Anopheles* mosquitoes representing all morphologically identified species demonstrated the presence of 20 distinct species (Table 3). The distinct sequence groups were arbitrarily named *Anopheles* species 1 through 20 (AN1 to AN20) prior to a more in-depth database comparison and species-level identification. ITS2 sequences from this study are available in GenBank (accession numbers PP537525–PP537544). Ten Cox1 sequences (GenBank PP587222–PP587231) were generated for clarification of ITS2-based sequenced identities. These 10 Cox1 sequences paired to 11 ITS2 sequence groups. Known specimens identified through ITS2 and Cox1 sequencing included *An. arabiensis* (*n* = 131; 19.76%), *An. pretoriensis* (*n* = 86; 12.97%), *An. rufipes* (*n* = 82; 12.37%), *An. cinereus* (*n* = 47; 7.09%), *An. christyi* (*n* = 46; 6.94%), *An. sergentii* (*n* = 42; 6.33%), *An. coustani* (*n* = 41; 6.18%), *An. pharoensis* (*n* = 30; 4.52%), *An. leesoni* (*n* = 7; 1.06%), *An. funestus* (*n* = 6; 0.90%), *An. nili* (*n* = 4; 0.60%), *An. maculipalpis* (*n* = 3; 0.45%), and *An. longipalpis C* (*n* = 2; 0.30%). Of the seven sequence groups that could not be identified to a specific species, the previously identified and sequenced *An.* sp. 1. BSL-2014 (*n* = 48; 7.24%) [25] was identified as *An. demeilloni* (Thomas Walker, London School of Hygiene and Tropical Medicine, pers. comm.). This species represented 24.1% of the collection in the highlands and 17.5% of the collection in the lowlands. *Anopheles fuscivenosus*, identified based on its sequence homology to *An. rivulorum*, was also previously described in Ethiopia [30] and identified morphologically as *An. fuscivenosus*. Members of the *An. coustani* (*An.* cf. *coustani*) and *An. pharoensis* (*An.* cf. *pharoensis*) complexes could not be identified to species. Notably, although the ITS2 sequences were different, the Cox1 sequences of the samples described as *An. pharoensis* and *An.* cf. *pharoensis* were identical, suggesting possible introgression or speciation in progress. The three remaining species groups were identified as the closest taxonomic group based on ITS2 and/or Cox1 homology. These included specimens belonging to the subgenus Nyssorhynchus, group Demeilloni, and series Neomyzomyia.

**Table 3:** Species identification based on ITS2 and/or Cox1 sequencing

Species group	ITS2 homology	Cox1 homology	Final species ID
AN1	*An. arabiensis*	–	*An. arabiensis*
AN2	*An.* sp. 1 BSL-2014	–	*An. demeilloni*
AN3	*An. christyi*	–	*An. christyi*
AN4	*An. cinereus*	–	*An. cinereus*
AN5	*An. coustani*	–	*An. coustani*
AN6	*An. funestus* sensu stricto (s.s.)	*An. funestus* s.s.	*An. funestus* s.s.
AN7	*An. leesoni*	–	*An. leesoni*
AN8	*An. longipalpis C*	*An. funestus*	*An. longipalpis C*
AN9	*An. maculipalpis*	–	*An. maculipalpis*
AN10	*An. nili*	–	*An. nili*
AN11	*An. pretoriensis*	*An. pretoriensis*	*An. pretoriensis*
AN12	*An. pharoensis*	*An. pharoensis*	*An. pharoensis*
AN13	*An. rufipes*	–	*An. rufipes*
AN14	*An. sergentii*^a^	*An. sergentii*	*An. sergentii*
AN15	*An* cf. *rivulorum*	*An* cf. *rivulorum*	*An. fuscivenosus*^a^
AN16	*An.* sp. isolate 10284–58^a^	*An. pharoensis*^a^	*An.* cf. *pharoensis*^a^
AN17	*An.* sp. O/15	*An. coustani*	*An.* cf. *coustani*^a^
AN18	*An. pulcherrimus*^a^	*An. oswaldoi*^a^	Subgenus *Nyssorhynchus*^a^
AN19	*An.* sp. 17 DZ-2020	*An.* sp. KHH1	Series Neomyzomyia^a^
AN20	*An.* sp. voucher 2022ETH0173	*An.* sp. NFL-2015	Group Demeilloni^a^

^a^Represents low similarity (below 98% for ITS2 and below 95% for Cox1) and/or unidentified species. Primary identifications were conducted using ITS2 sequencing, with Cox1 being utilized if the ITS2 sequence was not sufficient to identify the specimens to species

*AN Anopheles* species, *ITS2* internal transcribed spacer region 2, *Cox1* Cytochrome *c* oxidase subunit 1

### Biting behavior of *Anopheles* species

#### The highland villages

**Fig. 3:**
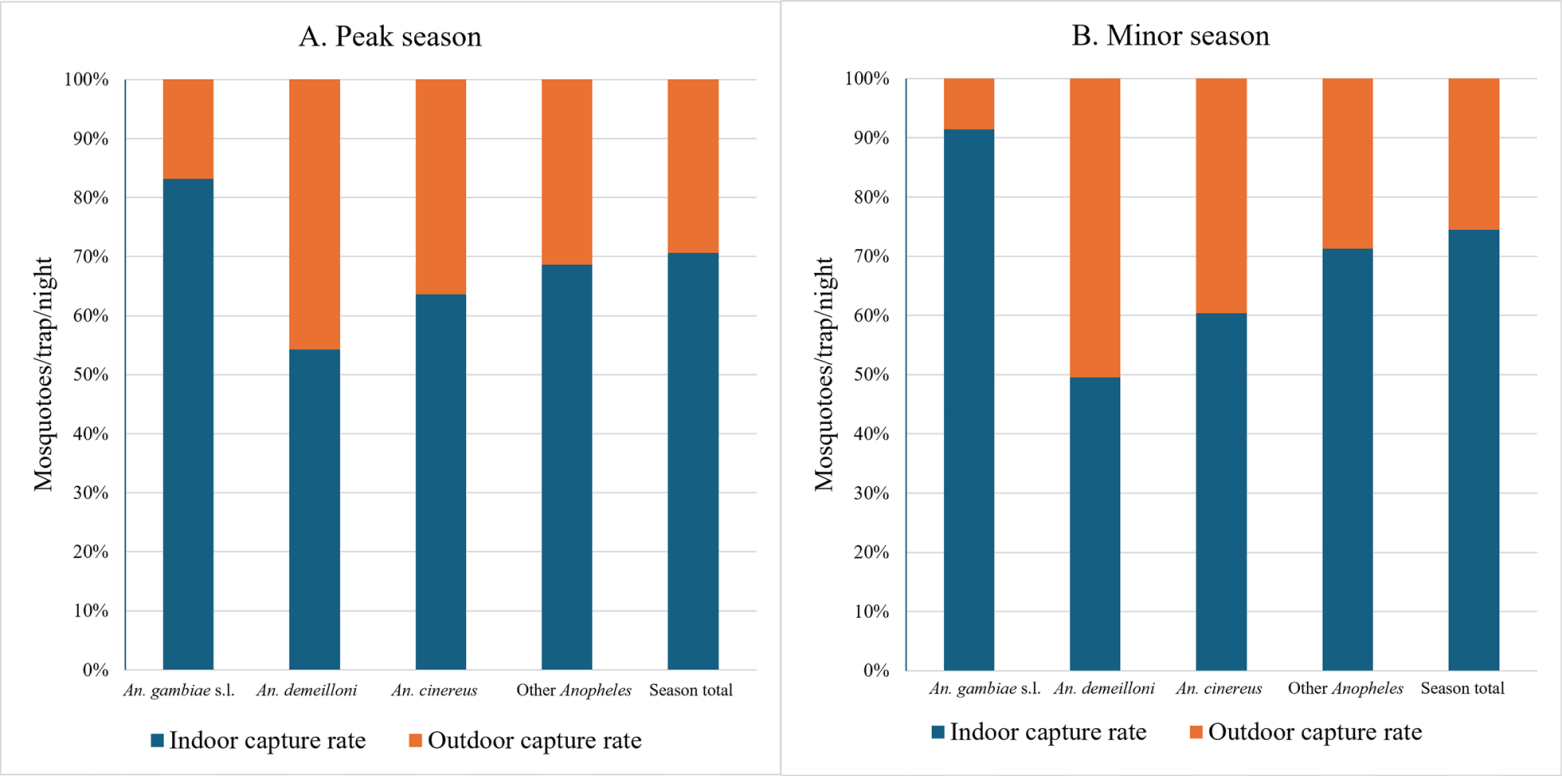
Indoor and outdoor capture rate in the highlands in both the (**A**) peak and (**B**) minor transmission seasons. The capture rates (mosquitoes per trap per night) are provided for the three most abundant species and all other *Anopheles* species together

Overall, *Anopheles* mosquitoes were more endophilic exhibiting indoor capture rates of 14 mtn and 5.83 mtn outdoors (*P* = 0.008) in the peak transmission season. This endophily was also seen in the minor transmission season with overall capture rates of 2.66 mtn indoors and 0.91 mtn outdoors. Of the three most abundant species, *An. gambiae* s.l. was the most endophilic, being captured 4.96 times more indoors (6.9 mtn indoors and 1.39 mtn outdoors) in the peak transmission season, while endophily increased in the minor transmission season, with 10.69 times more indoors (1.39 mtn) than outdoors (0.13 mtn). *Anopheles demeilloni* was captured at almost equal rates both indoors and outdoors in both peak and minor seasons. *Anopheles cinereus* was also documented as being more endophilic, with capture rates about 1.74 times and 1.53 times more indoors than outdoors in the peak and minor transmission seasons, respectively. All other species (combined) were also more endophilic than exophilic in both seasons (Fig. 3, Table 4).

**Table 4:** Nightly indoor and outdoor capture rates from the peak and minor transmission seasons for *Anopheles* species (morphological) from both the highlands and lowlands of northwestern Ethiopia

Site	*Anopheles* species (morphological)	Peak transmission season		Minor transmission season	
		Indoor collection mtn (*n*)	Outdoor collection mtn (*n*)	Indoor collection mtn (*n*)	Outdoor collection mtn (*n*)
Highland villages	*An. gambiae* s.l.	6.90 (1436)	1.39 (290)	1.39 (223)	0.13 (21)
	*An. demeilloni*	2.65 (552)	2.24 (466)	0.36 (57)	0.36 (58)
	*An. cinereus*	1.43 (297)	0.82 (170)	0.20 (32)	0.13 (21)
	Other *Anopheles* species	3.01 (627)	1.38 (287)	0.71 (114)	0.29 (46)
	Total *Anopheles*	14.00 (2912)	5.83 (1213)	2.66 (426)	0.91 (146)
Lowland—resident population villages	*An. gambiae* s.l.	3.03 (315)	1.99 (207)	1.36 (109)	1.99 (159)
	*An. pretoriensis*	2.26 (235)	3.21 (334)	1.45 (116)	1.99 (159)
	*An. demeilloni*	2.20 (229)	2.56 (266)	0.34 (27)	0.26 (21)
	Other *Anopheles* species	1.97 (205)	2.31 (240)	0.46 (37)	0.37 (30)
	Total *Anopheles*	9.46 (984)	10.07 (1047)	3.61 (289)	4.61 (369)
Lowland—seasonal migrant workers camps	*An. gambiae* s.l.	2.13 (222)	1.74 (181)	0.01 (1)	0.05 (4)
	*An. pretoriensis*	0.12 (12)	0.32 (33)	0.01 (1)	0.03 (2)
	*An. demeilloni*	0.12 (12)	0.08 (8)	0.00	0.01 (1)
	Other *Anopheles* species	0.22 (23)	0.27 (29)	0.03 (2)	0.00
	Total *Anopheles*	2.59 (269)	2.41 (251)	0.05 (4)	0.09 (7)

mtn = mosquitoes per trap per night, *n* = total collected

**Fig. 4:**
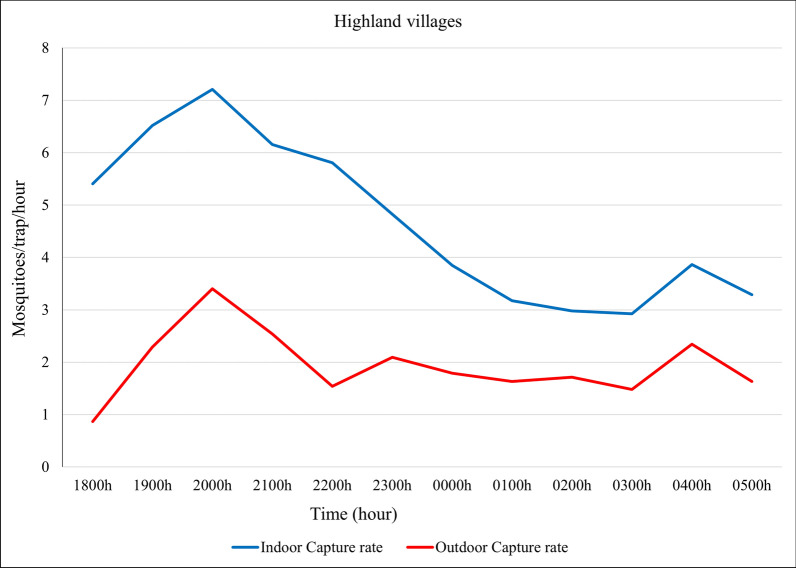
Indoor and outdoor overall *Anopheles* catch rates (mosquitoes per trap per hour) over the night in the four highland villages, northwestern Ethiopia

When looking at the time of capture, *Anopheles* mosquitoes were captured throughout the night, both indoors and outdoors, with an indoor peak occurring between 18:00 and 22:00 and an outdoor peak occurring between 19:00 and 21:00 (Fig. 4).

#### Lowlands: resident population villages

**Fig. 5:**
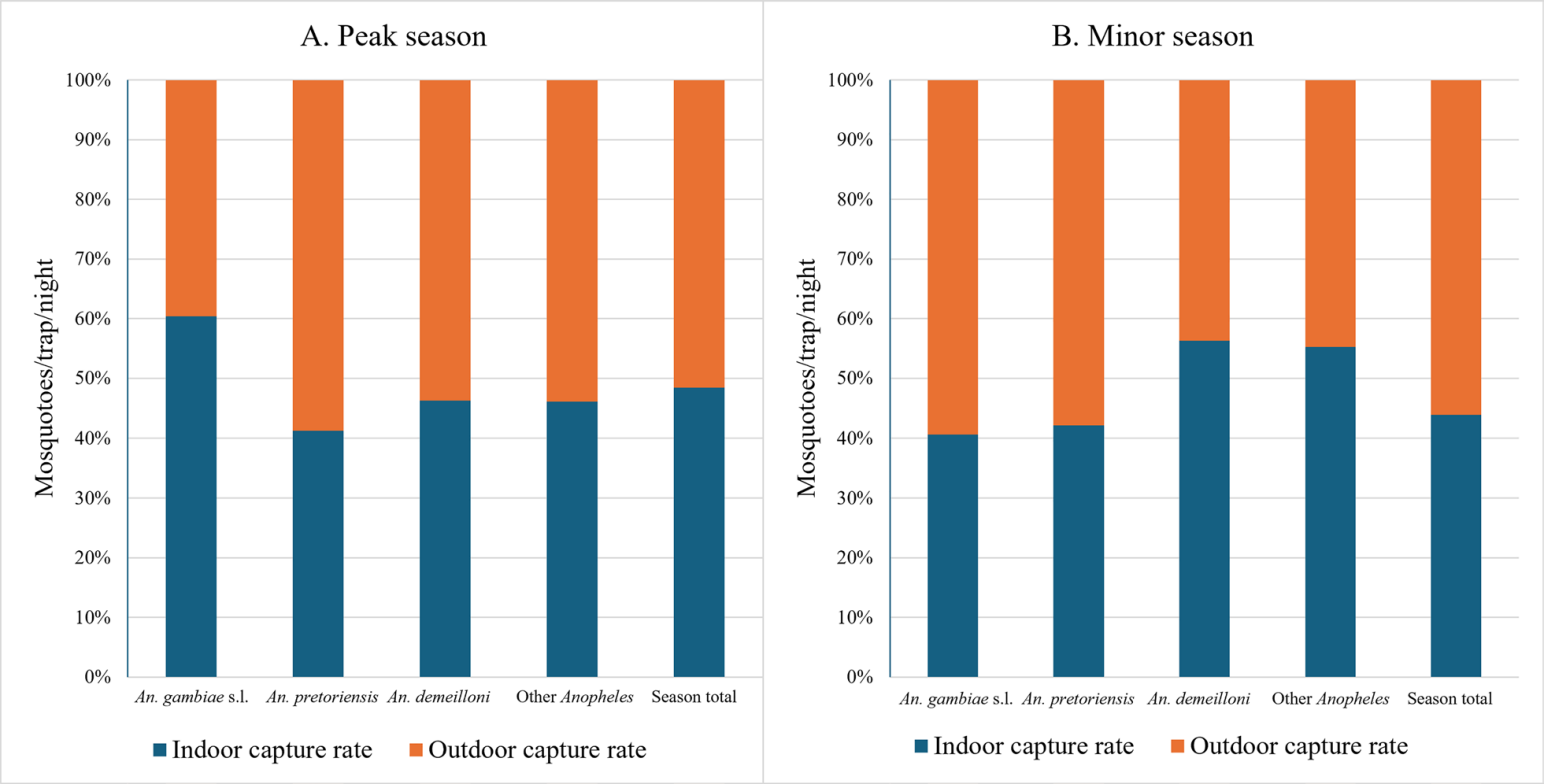
Indoor and outdoor capture rate in the lowland—resident population villages in both the (**A**) peak and (**B**) minor transmission seasons. The capture rates (mosquitoes per trap per night) are provided for the three most abundant species and all other *Anopheles* species together

In resident population villages*,* during both transmission seasons, *Anopheles* mosquitoes displayed greater exophily in both seasons: peak season (9.46 mtn indoors and 10.07 mtn outdoors) and minor season (3.61 mtn indoors and 4.61 mtn outdoors) (*P* = 0.03). *Anopheles gambiae* s.l. exhibited a seasonal shift in behavior. In the peak season, it was slightly endophilic, being captured 1.52 times more indoors (3.03 mtn indoors; 1.99 mtn outdoors). However, in the minor season, it became more exophilic, with capture rates about 1.39 times higher outdoors (1.89 mtn outdoors; 1.36 mtn indoors). *Anopheles pretoriensis* and *An. demeilloni* exhibited exophily in both the peak and minor transmission seasons. All other *Anopheles* species (combined) were also documented as being more exophilic with capture rates about 1.18 times and 1.38 times more outdoors than indoors in the peak and minor transmission seasons, respectively (Fig. 5**, **Table 4).

**Fig. 6:**
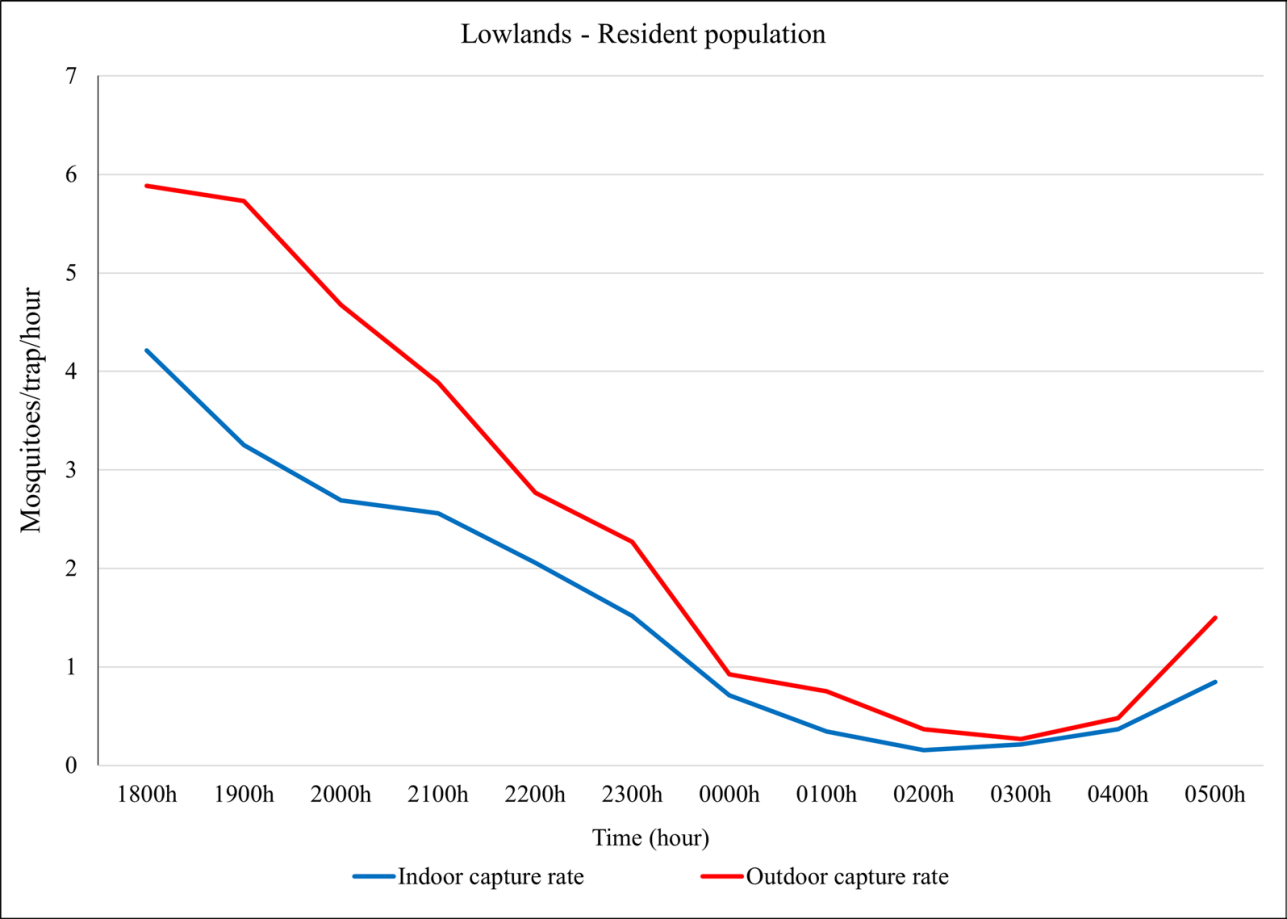
Indoor and outdoor overall *Anopheles* catch rates (mosquitoes per trap per hour) over the night in the lowland—in the resident population villages, northwestern Ethiopia

In the lowland resident population villages, *Anopheles* mosquitoes were captured throughout the night, both indoors and outdoors with peaks occurring between 18:00 and 21:00 both indoors and outdoors (Fig. 6).

#### Lowlands: seasonal migrant workers camps

**Fig. 7:**
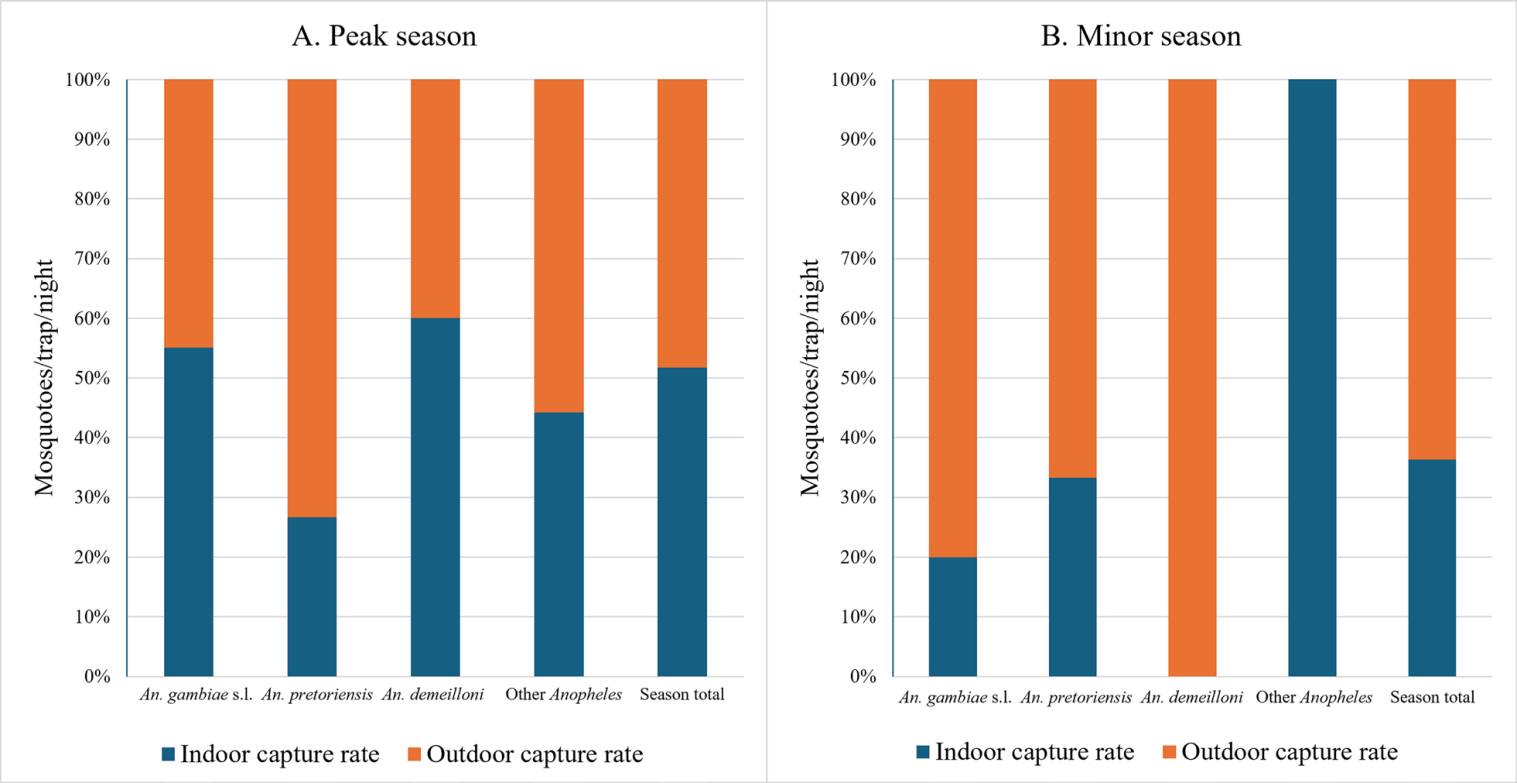
Indoor and outdoor capture rate in the lowland—seasonal migrant worker camps in both the (**A**) peak and (**B**) minor transmission seasons. The capture rates (mosquitoes per trap per night) are provided for the three most abundant species and all other *Anopheles* species together

Among the seasonal migrant workers, the pattern was similar to that of the villages of the resident population. Both *An. gambiae* s.l. and *An. demeilloni* were found indoors during the peak season and outdoors during the minor season, while *An. pretoriensis* showed a high preference for exophily in both seasons (Fig. 7**, **Table 4).

**Fig. 8:**
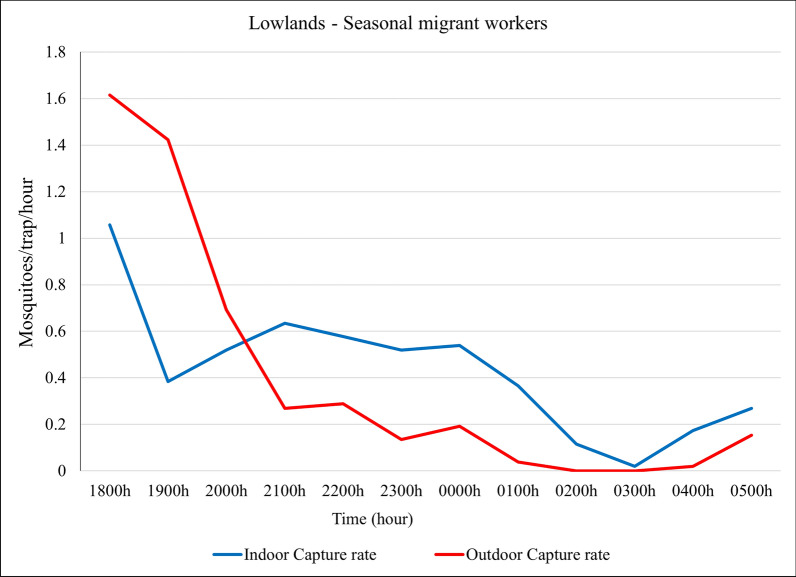
Indoor and outdoor overall *Anopheles* catch rates (mosquitoes per trap per hour) over the night in the lowland—in the camps of seasonal migrant workers, northwestern Ethiopia

In the lowland, at seasonal migrant workers camps, *Anopheles* mosquito peak indoor capture rate occurred between 18:00 and 19:00, and the outdoor capture rate peaked between 18:00 and 20:00 (Fig. 8).

### Seasonal variation in* Anopheles* species in the highlands and lowlands

Species composition and biting behaviors differed between the highlands and lowlands as well as between seasons. The highest *Anopheles* species diversity and abundance in almost all the study villages/camps were recorded during the peak transmission season. In the highlands, 87.8% (4125 of 4697) of the *Anopheles* mosquitoes were trapped during the peak malaria transmission season, whereas 79.2% (2551 of 3220) of the mosquitoes were trapped in the lowlands (Table 4).

## Discussion

The effectiveness of vector control strategies depends on the interaction and overlap between interventions and species-specific bionomic traits of local vector populations. Therefore, knowledge of vector compositions, density, seasonal variation, and behaviors [31] is vital for developing effective control strategies—in terms of intervention selection, timing of implementation, and expectations of impact. The absence of this baseline knowledgebase results in the blind implementation of a non-targeted strategy which usually results in continued and uncharacterized gaps in protection [32] and sustained local transmission. This study fills an important knowledge gap by investigating baseline entomological drivers of malaria transmission in resident and seasonal migrant worker populations in the lowlands and the source areas for migrant laborers in the highlands. Evidence generated characterizes highland and lowland *Anopheles* compositions along with bionomic traits that impact intervention effectiveness.

In the present study, *Anopheles* species composition and behaviors were quantified and described for both the highlands and lowlands (resident population and seasonal migrant workers) during the peak (at the end of the major rain, “*kiremt*”) and minor (at the end of the small rain, “*belg*”) malaria transmission seasons. Entomological surveys revealed variations in *Anopheles* species composition, abundance, and behavior in the highlands and lowlands. Molecular data demonstrated the presence of at least 20 *Anopheles* species in the lowlands and highlands of northwestern Ethiopia. *Anopheles arabiensis* (morphologically identified as *An. gambiae* s.l.) was the most abundant *Anopheles* species, suggesting that it is the principal malaria vector in both the highlands and lowlands [7, 18, 19]. The presence of *An. demeilloni, An. cinereus* [8], and *An. pharoensis* in the highlands, and *An. pretoriensis* and *An. demeilloni* in the lowlands point to complex entomological systems based on geography.

The species composition determined via morphological identification overlapped with the molecular results for the most abundant species in the area, i.e., *An. gambiae* s.l.*, An. pretoriensis*, and *An. cinereus*. However, molecular analysis revealed three *Anopheles* species (*An. funestus* group, *An. nili*, and *An. sergentii*) that were not initially identified via morphological methods. Moreover, sequencing also revealed various levels of misidentification based on the species. For example, sequencing-confirmed *An. arabiensis* was misidentified morphologically as four other species, with 69% of the identifications being accurate (morphologically identified as *An. gambiae* s.l.). Species (both known and unidentified) may have remained undescribed if morphology was the sole identification method used. Thus, these findings underscore the crucial role of molecular tools in complementing traditional methods towards understanding mosquito biodiversity. Moreover, since only 8.4% of all *Anopheles* were identified molecularly, it is possible that additional species are also present in this collection. Both morphological misidentification and the presence of novel species demonstrate the importance of molecular tools for species identification.

In the highlands, *An. gambiae* s.l., *An. demeilloni*, and *An. cinereus* were the most abundant species. The *Anopheles* mosquito fauna described here are similar to those observed in other highland geographies of Ethiopia [6–8]. The three most common species were followed by smaller numbers of *An. pharoensis*. As reported from different parts of Ethiopia, all four of these species transmit malaria, indicating that they probably contribute to local endemic transmission of malaria in the highlands of Ethiopia [7, 16]. Molecularly confirmed *An. arabiensis* is the predominant species recorded here and has long been identified as a primary malaria vector in Ethiopia with high rates of *Plasmodium* infection [15, 18]. Similarly, *An. demeilloni* has also been reported from highland sites, and was the second most common *Anopheles* species after *An. arabiensis* elsewhere in Ethiopia [7]. These findings are also in agreement with the findings from the western Kenya highlands, which reported *An. demeilloni* as the most dominant [25] and the second most dominant species after *An. christyi* [33]. *Anopheles cinereus* is another common species in the highlands, and its presence was previously documented in the highlands of Ethiopia [7, 8]. A report from a nearby village to the current study area indicated that *An. cinereus* was *P. falciparum* circumsporozoite protein (CSP)-positive [8], which was also reported in Eritrea [34].

In the lowlands, the current study revealed high *Anopheles* diversity. Eighteen different molecularly identified *Anopheles* species were collected from villages of the resident population and from seasonal migrant worker camps (Dellelo farm areas). This high species diversity may result from the presence of varied ecological and climatic factors favoring the larval development of different species [2, 6, 35]. About 78.4% of *Anopheles* mosquitoes were collected from Wedigemzo village. This village utilizes the nearby Guwang River and other seasonal rivers for irrigation alongside coastal sources, especially during the peak malaria transmission season. These small irrigation practices, along with puddles forming around the river's edge, might create an ideal habitat for mosquitoes. Studies from the different parts of Ethiopia support this link between small-scale irrigation and mosquito abundance [4, 18, 36]. A high number of *Anopheles* mosquitos corresponding to the presence of several vector species that may play either a primary or secondary role in the same area also significantly increases the risk of malaria transmission and might make malaria control more challenging. Thus, the control of these vector species may require the implementation of specifically tailored intervention strategies, including novel tools in addition to existing tools [18, 37]. This study suggested that malaria vector control interventions need to be strengthened in lowland villages to reduce the burden of malaria.

Morphologically identified *An. gambiae* s.l.*, An. pretoriensis*, and *An. demeilloni* were the three most common *Anopheles* species in the lowland villages. These findings accord with those of other studies from different parts of Ethiopia [15, 17–19]. A high number of *An. pretoriensis* were documented in the lowlands, especially in the resident villages. Although *An. pretoriensis* has not been implicated as a vector of malaria in Ethiopia, it is reported in the eastern, southwestern, and northern parts of the country [38–40]. A study from Zambia showed that *An. pretoriensis* was positive for *P. falciparum* [41], suggesting that understanding the contribution of this species to malaria transmission in Ethiopia is important. The presence of less common *Anopheles* mosquito species may require further investigation toward understanding human–vector contact and their potential role as vectors. In general, the diverse *Anopheles* species composition and abundance in both the highlands and lowlands highlight the importance of conducting routine entomological surveillance across the different parts of the country to monitor changes across time and location for better tailoring of interventions.

Understanding local vector behavior is important for evaluating their contribution to malaria transmission and providing guidance for the tailoring and targeting of interventions. In addition, the biting behavior of mosquitoes is an important risk factor for infection with malaria parasites [42]. With respect to species-specific vector bionomic traits, this study documented evening biting behaviors outdoors and early in the lowlands. This indicates that there might be a high risk to people working at night and an increased level of malaria transmission outdoors [9]. Thus, the primary interventions used for protection in the country, long-lasting insecticide-treated nets (LLINs) and indoor residual spraying (IRS), might fall short due to outdoor biting behaviors in the lowlands. In contrast, in the highlands, the three primary vectors, along with secondary vectors, indicate greater possible exposure indoors and early in the evening, with the temporality and intensity of exposure varying based on the density of the vector. Hence, malaria prevention and control measures should ideally factor in the spatial and temporal heterogeneity of exposure profiles [31]. Therefore, to choose the best mosquito control methods, local mosquito species and their behaviors (bionomics) must be considered, since these vary geographically and impact human exposure [31, 41]. In the lowlands, the outdoor early evening peak biting times of *An. gambiae* s.l. present a challenge for the protection of both seasonal migrant workers and resident populations from infectious bites. However, in the highlands, the rate of indoor capture of *An. gambiae* s.l. was much greater than that of outdoor capture, which indicates the importance of indoor biting for malaria transmission. Therefore, the vector interventions that work against this species in the highlands may not be as effective in the lowlands. Furthermore, the early evening peak biting activity in the highlands might render common vector control methods like LLINs less effective, as people may not be under the nets yet when the peak occurs. Therefore, while indoor interventions remain crucial, addressing these identified gaps in protection with additional interventions could significantly disrupt mosquito transmission.

In the present study, despite the variability in species composition and abundance throughout the collection period, *Anopheles* mosquitoes were detected throughout almost every month of collection. Seasonal variations were observed both in the highlands and lowlands, with an increase in mosquito populations following *kiremt* and a decrease toward *belg,* which may be related to the presence and abundance of larval habitats in the study areas. This finding is in line with previous observations from different parts of Ethiopia [4, 9, 17, 18]. It is important to note that the peak agricultural activities in both the highlands and lowlands coincided with the peak *Anopheles* mosquito densities, suggesting the economic significance of malaria*.*

## Conclusions

This targeted entomological surveillance in northwestern Ethiopia revealed crucial differences in mosquito diversity, behavior, and seasonality between the highlands and lowlands, necessitating tailored malaria control strategies based on the population being observed. While the highlands suite of *Anopheles* species primarily includes indoor biting, the lowlands boast diverse fauna, including the primary vector, *An. arabiensis*, which exhibits outdoor and early evening activity, potentially outsmarting conventional tools such as LLINs and IRS. Seasonal variations in mosquito abundance tied to rainfall patterns and the economic significance of the peak transmission period coinciding with agricultural activities further emphasize the need for targeted interventions. Routine entomological surveillance, spatially and temporally tailored control measures, and further investigations into secondary vectors are crucial for effectively managing malaria in this ecologically diverse region.

## Data Availability

Data supporting the study conclusions and outcomes of this article are included in the article. The ITS2 and Cox1 sequences supporting this study's findings have been deposited in GenBank under accession codes PP537525–PP537544 and PP587222–PP587231, respectively. The raw datasets presented and analyzed in this study are available upon request from the corresponding author.

## Abbreviations

CDC LT: Centers for Disease Control and Prevention light trap
Cox1: Cytochrome *c* oxidase subunit 1
IRS: Indoor residual spraying
ITS2: Internal transcribed spacer region 2
LLINs: Long-lasting insecticide-treated nets
masl: Meters above sea level
mtn: Mosquitoes per trap per night
s.l.: Sensu lato
